# Is Dentistry a Remunerative Profession?

**Published:** 1899-09

**Authors:** 


ARTICLE IV.

### IS DENTISTRY A REMUNERATIVE PROFESSION?

The close of the present term in all the dental colleges leads the thought in the direction of the graduating classes passing out to the great world of activity and becoming part of its working life. There is probably no duty of the conscientious professional teacher that carries with it a heavier burden than the anxiety as to the future of the hundreds of young men he has assisted to prepare for the competitive struggle that lies before them. This anxiety is comparatively of modern growth in dentistry, for the college, as late as 1876, that had ninety dental students in the school was regarded as eminently successful. While many of the smaller schools at present do not exceed this, others have four hundred as the normal number, and several have five hundred, with the prospective assurance of extending these limits.

The reason for this great influx into dentistry is not far to seek. The tendency to centralization of capital in every direction, while it may not, as often asserted, make the rich richer and the poor poorer, it does keep the young man of limited means in a subordinate position, bound to long hours of work, with a salary that will scarcely meet his own needs, much less enable him to assist in the maintenance of others. If gifted with extraordinary ability, he may hope eventually to rise, in the distant future, to the position of general manager, but this is so remote that a pessimistic spirit takes hold of his entire nature, and discouraged, he drops into the ruts of routine life in which he becomes simply a machine for capital to use and exhaust. This state of affairs has assumed serious proportions, and is bound to disturb, sooner or later, the economic conditions of the world. If capital does not create poverty, it at least does create a species of slavery in which men and women are bought and sold through certain hours of every day, and during these periods have no will of their own. This is not all evil, for the majority of workers, who are, perhaps, not qualified either by mental training or natural force to be leaders, drop, by mental gravitation, into subordinate places and are content.

This is, however, not the case with many who have been educated for higher things. The technical schools are turning out yearly great numbers of young men as civil, mechanical and electrical engineers, fitted to hold high positions, but who are forced to accept, for long periods, barely sufficient remuneration to keep life together. When, in after years, this may have been increased to the daily wages of the carpenter and bricklayer, it still means a perpetual struggle, and hope dies in the effort.

Parents have sounded the prospects, to some degree, of these various callings, and have hesitated to send sons to institutions for four years, at great cost, to find, in the end, every place crowded.

The result has been that parents, whether wisely or unwisely, have determined for themselves that that profession is the best which for the least outlay of capital, will furnish an independent livelihood, and this they deem to exist in dentistry and medicine, preferably the former, for it requires, after graduation, less time to become self supporting. The instinct of parents—for it can scarcely be called knowledge—is mainly correct. The stomatologist occupies a truly independent position, and while his income will rarely reach that of the successful practitioner in medicine, he is more comfortably fixed, in that he not only controls his life-work, but is far more independent as to hours of labor.

This brings prominently before the mind the thought, is dentistry, as practiced to-day, a remunerative calling? If this were not so it would scarcely be worth the labor that is being devoted to it in the educational sense, for whatever may be thought of dental educational methods and institutions, and they are constantly subjected to severe criticism, they are not turning out dentists by the wholesale without an anxious regard to their future well-being.

The writer of this has made this subject one of careful study, and has become convinced that the growth of knowledge upon dental subjects by the people at large fully warrants the apparent over production of dentists. The care of the teeth has ceased to be luxury of the wealthy, but is eagerly sought by those of moderate means, and even the very poor crowd our college dispensaries. In fact, at no period has the value of dental service been more thoroughly appreciated. The lessons so laboriously taught by the pioneers in the profession are bearing rich fruit, and to meet the demand of these occasions there must be a continued and increasing development of skilled men and women.

This means a gradually decreasing clientage for each practitioner. In the earlier day it was in proportion to population, one dentist to each seven to ten thousand inhabitants; now, if the next census gives seventy-five million inhabitants to the United States, and we have twenty five thousand dentists—Polk's Register says over twenty thousand—we have this reduced to three thousand to each practitioner. While this seems to be a serious reduction, it is more apparent than real, for the higher dental education of the laity amply makes up for the loss of numbers upon the financial side, and more than compensates for this loss by the intelligent appreciation of the work, which to the true stomatologist is more than money.

It is true that dentistry, as a money-making profession, is not equal to others. Taking the country through, it is questionable whether, from the increase of the profession, it would average two thousand dollars a year. This means but small savings, for expenses grow, with alarming proportions, as practice increases. It is thought, however, that while it requires more capital to-day to begin the practice of dentistry than it did forty years ago, that capital is well expended, for it gives greater facilities for accomplishing the work, and consequently increased ability to earn with far less labor.

It has been a current thought in our literature that all a dentist leaves his family is a life insurance policy. This was largely true in former years, but dentistry has been placed upon an altogether different basis, and the more the true professional spirit is infused into practitioners the more will this be increased. Dentists must learn to enforce a proper remuneration for services rendered. This should be rated according to the value these are to the patient and the reputation of the operator. There is too much of the mechanical or shop-keeping idea extant, of remuneration at so much per tooth. The operater should be paid for his time, independent of all expense to which he may be subjected.

It is difficult to understand the many life wrecks in the history of dentistry. Indeed, these, in the writer's memory, exceed the successes. The number of the latter, however, in recent years, enforces the belief that with the change of methods of practice has come a more economic arrangement of the time and means, and that in the near future there will be fewer of those unfortunate examples to disturb the thought of those entering professional life. Dr. Allport, of Chicago, was one of those who combined professional excellence with business methods. He publicly deplored, upon a certain occasion, that so few dentists husbanded their means for the period when old age would render them incapable of work. There seems to be no reason why a dentist should fail to accumulate a reasonable sum directly from professional labor. The temptation to some is to enter into hazardous speculation, for which they are poorly fitted, or to take up with side issues which always detract from their ability; in fact, this division of interest invariably tends to lower their work, and it becomes purely mechanical, the professional dying out altogether.

The conclusion is inevitably borne out by the facts, that dentistry is not overcrowded; that there is room for all these young graduates, provided they exhibit a marked aptitude for their calling; and, further, that an economical administration of practice will result in ample means for comfort, and even luxury, and, at the end, something will be left for those dependent upon them. This is more than, it is feared, can be accomplished by those in other branches of professional work. —International Dental Journal.

